# What is the effect of preterm birth on permanent tooth crown dimensions? A systematic review and meta-analysis

**DOI:** 10.1371/journal.pone.0259293

**Published:** 2021-11-05

**Authors:** Shaima Nasser Buhamer, Eleftherios Kaklamanos, Mawlood Kowash, Iyad Hussein, Anas Salami, Manal Al-Halabi

**Affiliations:** 1 Dubai Health Authority, Dubai, United Arab Emirates; 2 Hamdan Bin Mohammed College of Dental Medicine, Mohammed Bin Rashid University of Medicine and Health Sciences, Dubai, United Arab Emirates; University of Zurich, SWITZERLAND

## Abstract

**Background:**

The permanent tooth formation process may be disrupted in preterm infants with potential discrepancies in size and subsequent occlusal disturbances.

**Objective:**

To systematically analyse and quantitively synthesize the available evidence regarding the impact of preterm birth on permanent tooth crown dimensions.

**Search methods:**

Unrestricted searches in 6 databases and manual searching of the reference lists in relevant studies were performed up to March 2021 (Medline via PubMed, CENTRAL, Cochrane Database of Systematic Reviews, Scopus, Web of Science, ProQuest Dissertations and Theses Global).

**Selection criteria:**

Observational studies investigating permanent tooth crown dimensions in preterm and control full-term born individuals.

**Data collection and analysis:**

Following study retrieval and selection, relevant data were extracted, and the Newcastle-Ottawa scale was used to assess the selection, comparability, and outcome domains. Exploratory synthesis and meta-regression were carried out using the random effects model.

**Results:**

Three studies were located from the initially retrieved records and the assessments with the Newcastle-Ottawa scale identified issues regarding the selection and comparability domains. Overall, the mesiodistal and the buccolingual dimensions of the permanent teeth in both dental arches tended to be smaller in children born prematurely than full term children. Subgroup analyses showed statistically significant differences for the extremely preterm to control group comparisons for the incisors and the first molars. Meta-regression showed a modificatory effect of gestational age and racial background but not of birth weight and gender on tooth size. The quality of available evidence was rated at best as moderate.

**Conclusions:**

Premature birth could potentially be associated with reduced tooth-crown dimensions in some permanent teeth especially in children born extremely preterm. Although the results from these observational studies should be approached with caution until more information becomes available, the possible clinical implications in terms of diagnosis and treatment planning should be considered.

**Registration:**

PROSPERO (CRD42020182243).

## Introduction

Preterm infants are defined by the World Health Organization (WHO) as: “the babies born alive before 37 gestational weeks (GW) of pregnancy are completed” [1]. Worldwide, preterm birth rates are increasing with an estimated 15 million babies prematurely born per year [2]. Multiple risk factors are associated with the occurrence of preterm births, including race, socioeconomic status, and maternal age. A vast difference is noticed in the incidence of preterm births between the populations of the developing countries that can be attributed to the different living conditions provided [3]. Preterm infants are poorly equipped for the extrauterine life and usually need considerable medical intervention during the neonatal period. Serious complications are encountered in almost all the major organ systems including birth asphyxia, apnoea, hyaline membrane disease, patent ductus arteriosus, intracranial haemorrhage, renal immaturity, metabolic dysfunction, gastrointestinal intolerance, and high susceptibility to infections [4].

According to WHO, preterm infants may also be classified according to their weight independent of gestational age into Low Birth Weight (LBW) < 2500g; Very Low Birth Weight (VLBW) < 1500g; and Extremely Low Birth Weight (ELBW) < 1000g [5]. Neonates weighing less than 2500g are described as low birthweight infants. Low birth weight could be a result of preterm birth or restricted intrauterine growth. Neonates with low birth weight could be mature but small for gestational age [6]. Preterm birth is the most frequent cause of low birth weight [3].

Under normal conditions, the time, sequence and chronology of tooth formation, calcification and eruption follow a regular cycle. This process may be disrupted in preterm infants by exposure to certain medicaments and/or traumatic oral manipulations [7, 8]. The shorter the gestational age, the higher the risk for morbidity and medical complications as well as disruption of the biological tooth formation process that might lead to alterations in tooth crown size and the eruption [9]. Other tooth anomalies can be detected in children born prematurely. A recent meta-analysis concluded that there is a three times increased risk of developing developmental defects of the enamel in preterm children [10].

From the orthodontic treatment perspective, disproportions between permanent teeth dimensions and the size of the jaws might result in variations in the space available for alignment within the dental arches and the approach to treatment. Furthermore, abnormalities in permanent teeth size might lead to discrepancies in the upper to lower tooth size ratios. When permanent teeth are not matched for size, normal occlusion might be compromised [11]. A previous systematic review reporting on the relevant literature up to 2002 identified only one study on the permanent dentition with contradictory results on whether premature birth causes altered tooth crown dimensions [12].

### Objective

The aim of the present study was to systematically investigate for the most contemporary available information and quantitatively synthesize the available data regarding the effect of preterm birth on permanent tooth crown dimensions.

## Materials and methods

### Protocol and registration

The present review was based on a protocol developed, registered, carried out and reported following relevant methodological guidelines, including the PRISMA 2020 statement (PROSPERO: CRD42020182243) [13–17]. As the present study is a systematic review, ethical approval was not required.

### Eligibility criteria

The Population, Exposure, Comparator and Outcomes domains were used to describe the eligibility criteria (S1 Table) [18]. We looked for observational studies investigating permanent tooth crown dimensions in preterm and control full-term born individuals (as judged by the gestational age retrieved from medical/hospital records). We followed the World Health Organization definition for preterm birth as any birth before 37 completed weeks of gestation [19]. Since tooth size is determined early and definitely, no restriction was placed on the age of the study population; however, only studies involving individuals with mixed or permanent dentition were included. Moreover, we included individuals of any gender and racial background. The included studies had to verify gestational age at birth by medical/hospital records and report on the mesiodistal and/or buccolingual dimensions of permanent teeth (along with measurements of dispersion). Studies that did not include a comparison to a control group of full-term born children were excluded. Finally, we did not consider animal, in vitro, ex-vivo or in silico studies; non-comparative studies, reviews, systematic reviews and meta-analyses.

### Information sources and search strategy

One author (EGK) developed the detailed search strategies for each of the databases (including grey literature) that we searched until March 14^th^ 2021 (Medline (PubMed), CENTRAL (Cochrane Library; includes records from Embase, CINAHL, ClinicalTrials.gov, WHO’s ICTRP, KoreaMed, Cochrane Review Groups’ Specialized Registers, and records identified by handsearching), Cochrane Database of Systematic Reviews (Cochrane Library), Scopus, Web of Knowledge (including Web of Science Core Collection, KCI Korean Journal Database, Russian Science Citation Index, SciELO Citation Index and Zoological Record) and ProQuest Dissertation and Theses (ProQuest)) (S2 Table). We did not impose any restrictions on the language or date of publication. Duplicates were removed using EndNote’s duplicate identification strategy (EndNote X9™, Clarivate™, Philadelphia, PA, USA) and then manually by EGK. We also searched manually the reference lists in relevant article to identify additional studies (SB and EGK).

### Selection process, data collection process and data items

Two researchers (SB and EGK) assessed the titles and abstracts of all retrieved records for inclusion independently. In case of disagreement, consensus on which articles to read in full-text was reached by discussion. Subsequently, the researchers independently screened the full-text papers for potential inclusion. Again, in case of disagreement, consensus was reached by discussion. Finally, the authors used a predetermined form to extract data from the included studies. Extracted data were compared, and any discrepancies were resolved by discussion. The following information was extracted: bibliographic information, study design and eligibility; inclusion and exclusion criteria; population characteristics (numbers of study participants, gender, gestational age, birth weight; health status); outcome assessment (teeth measured, methods of measurement, dimensions assessed, power calculations, method error assessment); numerical results and information regarding the risk of bias assessment domains. The characteristics of the population under study and the numerical results were extracted separately for the exposed and unexposed group (in the case of a cohort study) or for the groups of cases and controls (in the case of a case-control study). For the cohort studies, the datasets were categorized on the basis of the gestational age to the following groups: extremely preterm group (EPT; birth before completed 28 weeks of gestation), very preterm group (VPT; birth at completed 28 weeks of gestation but before completing 32 weeks), and moderate preterm (MPT; birth at completed 32 weeks of gestation but before completing 37 weeks) [19, 20]. Since the African racial background has been associated with decreased gestational length by about one week [21–26] the above cut-off points were reduced likewise for children with such background following previous investigations [21, 26]. If clarifications were needed regarding the published data, or additional material was required, then attempts to contact the corresponding authors through email would be made.

### Study risk of bias assessment

The Newcastle-Ottawa scale was used to assess the selection, comparability, and outcome domains independently by SB and EGK [27–29]. In all the above-mentioned processes, disagreements were settled by discussion; kappa statistics were not calculated as it is not recommended any more in relevant guidelines [15].

### Effect measures and synthesis methods

Data on teeth dimensions are continuous. The random-effects method for meta-analysis was used exploratorily to compare the permanent teeth crown dimensions [mesiodistal (MD) and buccolingual (BL)] for each tooth between preterm and full-term children in appropriate statistical forms [weighted mean difference (WMD)] together with 95% confidence interval (CI). For syntheses including more that 10 datasets, the corresponding 95% prediction intervals were calculated also [15]. Comparisons between the three categories of preterm (MPT, VPT and EPT) and full-term children were conducted as well. To identify the presence and the extent of heterogeneity between studies, the overlap of 95% confidence interval for the results of individual studies was inspected graphically, and the I^2^ statistic was calculated [15]. All analyses were carried out with Comprehensive Meta-Analysis Software version 3 (©2014 Biostat Inc., Englewood, New Jersey, USA). Significance (a) was set at 0.05, except for 0.10 used for the Q test [30].

### Certainty assessment and additional analyses

As per protocol, analyses were to be carried out for “small-study effects” and publication bias but were not performed finally due to the lack of an adequate number of studies [15]. Where possible, subgroup analyses comparing the differences in permanent teeth dimensions in the three categories of preterm individuals (MPT, VPT and EPT) and the children of the control groups were carried out [15]. We used meta-regression to explore in univariate regression models whether gestational age, birth weight, racial background and gender modified the results. Subsequently, we incorporated any statistically significant variable (gestational age, birth weight, racial background and gender) into multivariate regression models. Finally, despite the lack of extensive information for some outcomes, the quality of the available evidence regarding the differences in the permanent teeth dimensions between preterm and full-term children was assessed with the Grades of Recommendation, Assessment, Development, and Evaluation in order to adopt a structured and transparent approach in formulating an interpretation of the evidence [28, 31].

## Results

### Study selection

**Fig 1:**
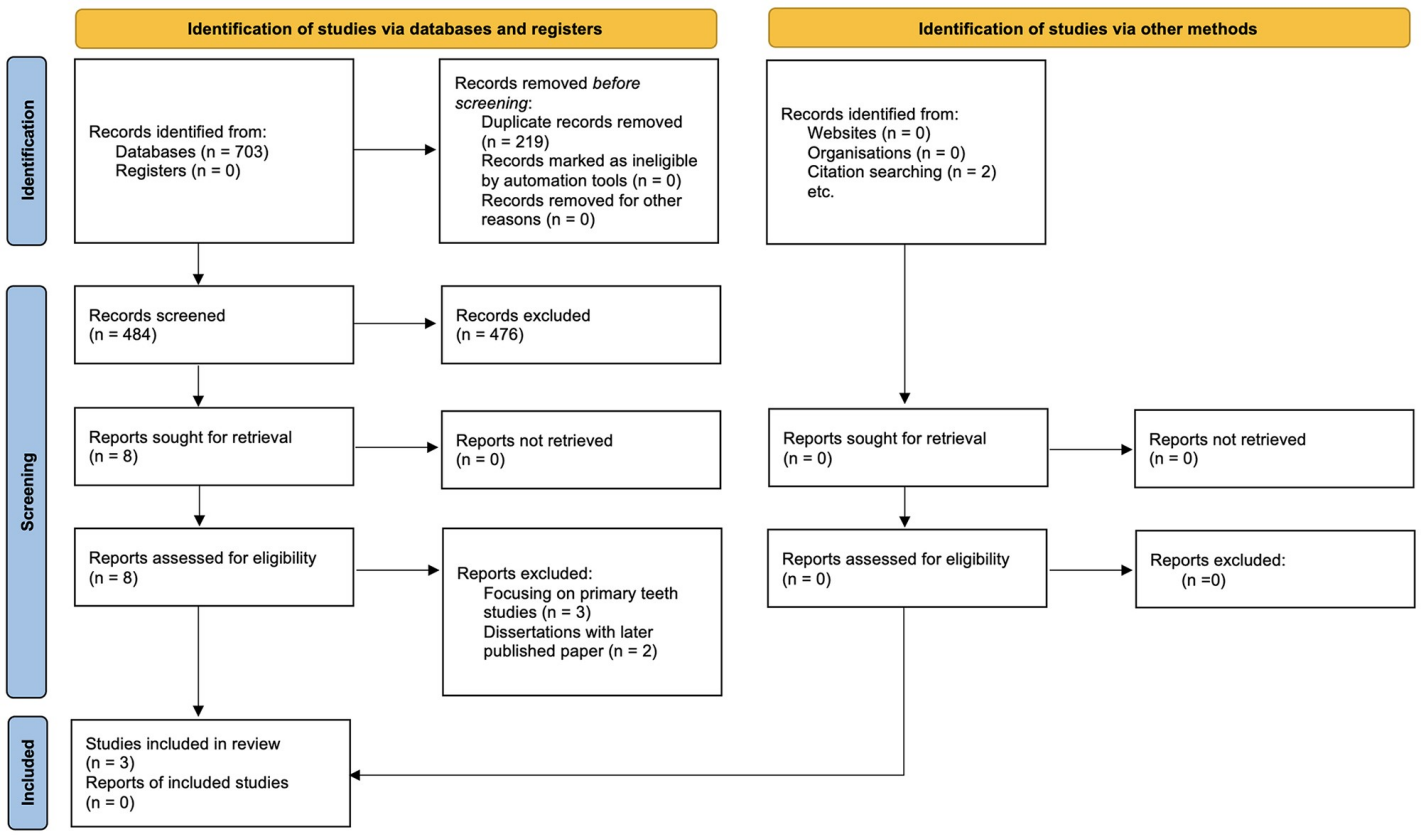
PRISMA 2020 flow diagram.

Seven hundred three records were found from databases searching and 2 more manually. After duplicates removal, 484 records were screened from which 8 were reviewed in full-text. Five records were excluded for the following reasons: three were studying primary teeth dimensional changes only [32–34] and the other two were dissertation publications based on which a paper was later published [35, 36]. Finally, 3 papers including 18 datasets were eligible for the review (Fig 1) [37–39]. Two of these papers were part of dissertation projects that were checked whether they included useful information additional to that in the published article [35, 36].

### Study characteristics

All finally eligible studies were of a cohort design; permanent tooth crown dimensions were assessed in preterm and full-term children and comparisons were made between them (Tables 1 and 2). They were published between 2001 and 2017 and did not include sample size calculations.

**Table 1:** General characteristics in the studies included in the systematic review.

Study	Inclusion and exclusion criteria	Population characteristics*
**Harila-Kaera et al**. [37] 2001 United States	**Inclusion criteria**: Caucasian/African American; 5–14 years of age; participants in the Collaborative Perinatal Study of the National Institute of Neurological Disorders and Stroke; dental examinations performed in the 1970s (Buffalo, NY; Richmond, VA; Portland, OR; Philadelphia, PA; Providence, RI; Johns Hopkins, MD)	**MPT Caucasian Group**: 60 [40 M, 20 F]
		**Gestational age (w)**: M 33.7 ±3.4, F 34.6 ±1.7
		**PT African American Group**: 278 [140 M, 128 F]
		**Gestational age (w)**: M 31.7 ±3.0, F 32.2 ±3.0
	**Especially for PT Group**: the limit of prematurity was placed at 36 weeks for Caucasians and 35 weeks form African Americans, since the average period of gestation is sorter in the latter	**FT Caucasian Group**: 803 [408 M, 395 F]
		**Gestational age (w)**: M 40.4±1.9, F 40.6 ±1.9
	Groups were not matched for age because of the early and definite determination of tooth size.	**FT African American Group**: 1001 [477 M, 524 F]
		**Gestational age (w)**: M 39.8 ±2.6, F 39.9 ±2.4
**Rythén et al**. [38] 2013 Sweden	**Inclusion criteria**: adolescents; born from 1988 to 1991 at the Sahlgrenska University Hospital in the city of Gothenburg [as registered in the Medical Birth Register], County of Västra Götaland, Sweden; subjects agreeing to participate.	**EPT Group**: 40 [25 M, 15 F]^1^
		**Gestational age (w)**: 27.4 ±1.06
		**Birth weight (gr)**: 1006 ±232
	**Especially for PT Group**: born before a gestational age of 29 weeks (total 56 infants).	**FT Group**: 40 [25 M, 15 F]^2^
	**Especially for FT Group**: born at term; individually matched, by age, gender and living area.	**Gestational age (w)**: 40 ±1.4
		**Birth weight (gr)**: 3585 ±391
**Ebrahim and Paulsson** [39] 2017 Sweden	**Inclusion criteria**: Caucasian; 8–10 years of age; born from 1992 to 1996 in the County council of Skane [as registered in the Medical Birth Register]; born at the University Hospitals of Lund and Malmö; living in the southwest part of the County council of Skane; subjects agreeing to participate.	**EPT Group**: 36 [25 M, 11 F]
		**Gestational age (w)**: 26.8 ±1.0
		**Birth weight (gr)**: 939.5 ±241
	**Exclusion criteria**: Children with syndromes or with neuromuscular disorders (e.g., cerebral palsy).	**VPT Group**: 37 [20 M, 17 F]
	**Especially for PT Group**: born in gestation weeks 23–32; in the EPT group, 56 children fulfilled the inclusion criteria and were invited to participate; in the VPT group, 184 children fulfilled the inclusion criteria and 52 of them were randomly selected and asked to participate.	**Gestational age (w)**: 30.8 ±1.1
		**Birth weight (gr)**: 1639.6 ±341
		**FT Group**: 41 [22 M, 19 F]
	**Especially for FT Group**: normal birth weight; matched for gender, nationality, month of birth and living area.	**Gestational age (w)**: 39.8 ±1.0
		**Birth weight (gr)**: 3581.2 ±470

EPT; Extremely preterm (birth before completed 28 weeks of gestation); F: female; FT: full term; L: Mandibular teeth; M: male; PT: preterm; U: Maxillary teeth; VPT: Very preterm (birth at completed 28 weeks of gestation but before completing 32 weeks); w: weeks

*Mean ±Standard Deviation;

^1^EPT Group children with dental cast measurements: 36 [22 M, 14 F];

^2^FT Group children with dental cast measurements: 39 [25 M, 14 F].

**Table 2:** Outcome measurement characteristics of the studies included in the systematic review.

Study	Teeth measured	Method of measurement and dimensions assessed	Additional information
**Harila-Kaera et al**. [37] 2001 United States	**U & L 1s, 2s & 6s** Teeth with attrition, decay or filling at the measurement points were not measured	**Measurements on study casts with digital calipers**	**Power calculation**: nm
		**Mesiodistal dimension**: maximal dimension parallel to the occlusal and labial surfaces	**Method error**: Dahlberg’ formula
		**Buccolingual dimension**: [6s only] maximal dimension in a plane perpendicular to the occlusal and labial surfaces	
**Rythén et al**. [38] 2013 Sweded	**U & L 1s, 2s, 3s, 4s, 5s, 6s & 7s**	**Measurements on study casts with digital calipers**	**Power calculation**: nm
		**Mesiodistal dimension**: the greatest distance between the contact points of each tooth	**Method error**: Dahlberg’ formula
**Ebrahim and Paulsson** [39] 2017 Sweden	**U & L 1s, 2s & 6s**	**Measurements on study casts with digital calipers**	**Power calculation**: nm
	Teeth missing or broken or not fully erupted were not measured. In the total sample, 49 teeth (41 2s, 6 6s, and 2 1s) of 1368 were not possible to measure.	**Mesiodistal dimension**: maximal distance between the contact points of each tooth parallel to the labial and occlusal surfaces	**Method error**: Dahlberg’ formula; Intraclass Correlation Coefficient
		**Buccolingual dimension**: [1s & 2s] the greatest distance between the cingulum and a point parallel to it buccally on the cervical line; [6s] greatest distance between the buccal groove and a point parallel to it lingually on the cervical line.	

L: Mandibular teeth; U: Maxillary teeth; 1s: central incisors; 2s: lateral incisors; 3s: canines; 4s: first premolars; 5s: second premolars; 6s: first molars; 7s: second molars.

The Harila-Kaera et al. study [37], which was conducted in the United States of America, categorized children according to gender, as well as their racial background into Caucasians and African Americans. The children were participating in the Collaborative Perinatal Study of the National Institute of Neurological Disorders and Stroke in the 1960s and the dental examinations performed in the 1970s at 6 calibrated centres. Although it is mentioned that prospective medical background data were obtained from the first registration of the pregnancy up to seventh year of age, including anamnestic information on the mother’s health and background (21), such information was not reported. The limit of prematurity was placed at 36 weeks for Caucasians and 35 weeks form African Americans, since the average period of gestation was found to be sorter by about one week in the latter group [21]. The populations under study were not matched for age, because of the early and definite determination of tooth size, nor for any other parameter.

The Rythé et al. study [38] was conducted in Swedish children born from 1988 to 1991. The study population was further subdivided according to gender. The neonatal and postnatal medical history, gestational age and birth weight were retrieved from hospital medical records for the preterm children and from the Swedish Medical Birth Registry for the controls. The preterm infants were hospitalized for a variety of time periods and treated with artificial ventilation, blood transfusions, antibiotics, artificial nutrition and in some cases, surgery. At the time of the clinical examination, 24 of the examined adolescents born preterm suffered from one or more medical diagnoses including chronic respiratory disease, heart disease, allergies, growth deficiencies, cerebral palsy, hearing, and visual defects. Neuropsychiatric disturbances were found in 11 children. One girl was diagnosed with Turner mosaicism and one girl with Pierre Robin syndrome. The control group children were individually matched for age, gender and living area with the preterm children. None of them had neonatal or postnatal medical diagnoses causing prolonged hospitalization and were healthy at the time of the clinical examination, except two who had allergies.

Like the previous investigation, Ebrahim and Paulsson [39] conducted their study in Swedish children born from 1992 to 1996; the children were subdivided according to gender. Individuals with syndromes or with neuromuscular disorders (e.g., cerebral palsy) were excluded. Gestational age and birth weight were retrieved from the Swedish Medical Birth Registry. The control group children were individually matched for matched for gender, nationality, month of birth and living area with the preterm children.

### Risk of bias in studies

**Fig 2:**
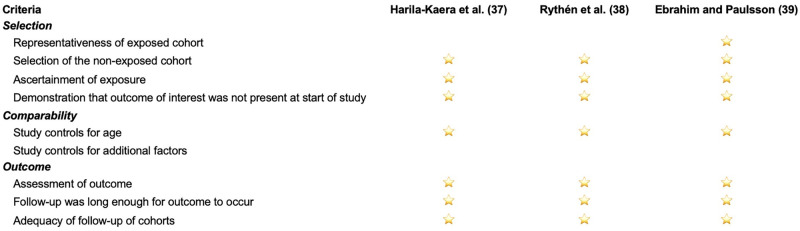
The Newcastle-Ottawa scale.

The Newcastle-Ottawa scale assessments are presented in Fig 2. The Harila-Kaera et al. study [37] involved children born in the 1960s, when medical care was not as advanced as today and babies of small gestational age and weight at birth exhibited a higher rate of mortality [40]. Both Harila-Kaera et al. [37] and Rythn et al. [38] considered all premature children, irrespective of health status and did not exclude infants with syndromes or with neuromuscular disorders (e.g., cerebral palsy), like Ebrahim and Paulsson [39]. In all three studies records regarding gestational age, health status at birth, gestational weight and post-natal morbidity were collected from hospital records. Regarding comparability, although important confounders like gestational age, birth weight, gender and racial background were considered, other genetic, epigenetic, and environmental influences, like maternal age and health, postnatal complications, and care, etc. that may affect individual development were not appropriately measured or were not controlled.

### Effect on permanent teeth dimensions

**Fig 3:**
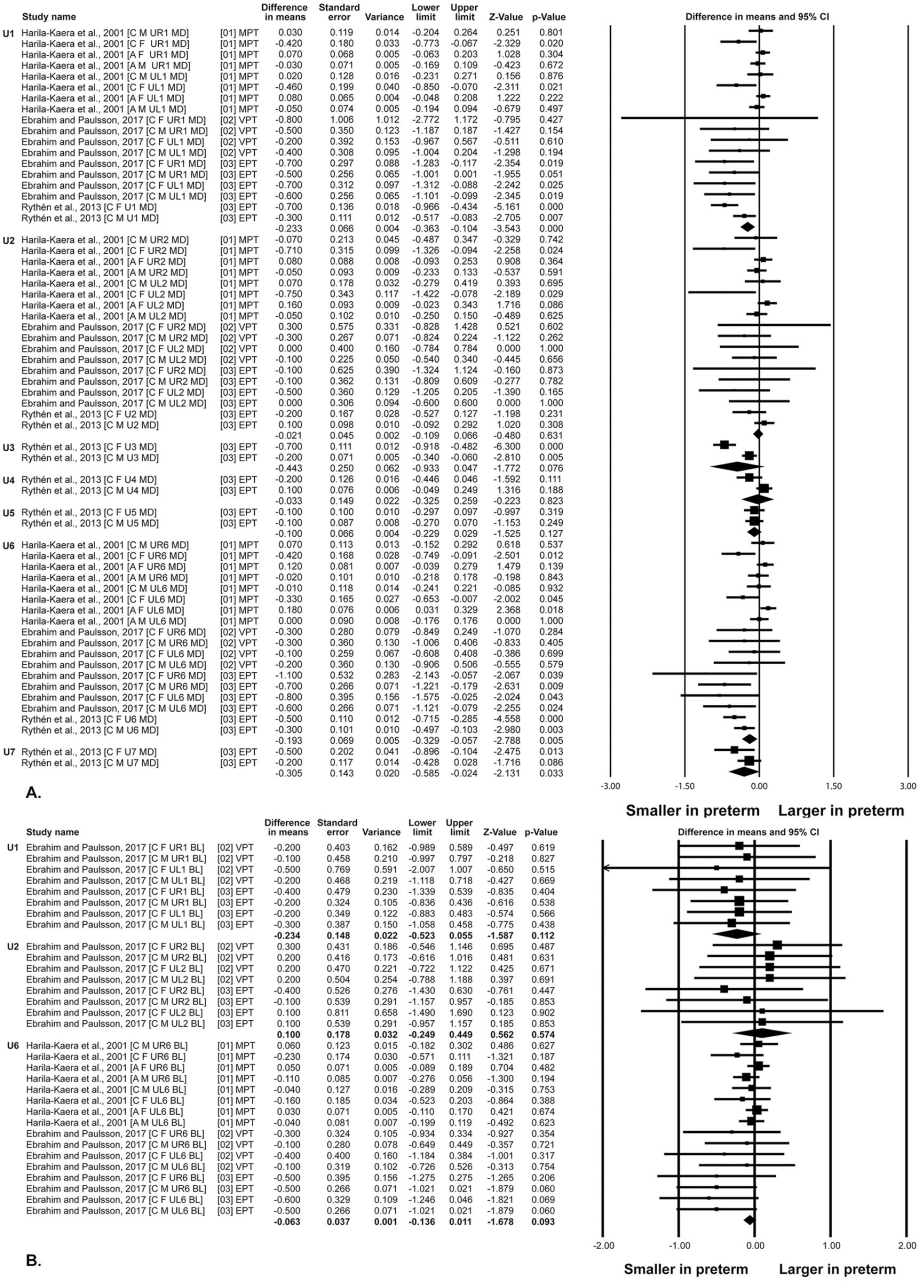
Differences in teeth dimension between preterm and control children. Maxillary teeth. A. Mesiodistal dimensions. B. Buccolingual dimensions. A: African American; BL: Buccolingual; C: Caucausian; CI: Confidence Interval; EPT; Extremely preterm; F: Females; M: Males; MD: Mesiodistal; MPT: Moderate preterm; UL: Maxillary left teeth; UR: Maxillary right teeth; VPT: Very preterm.

**Fig 4:**
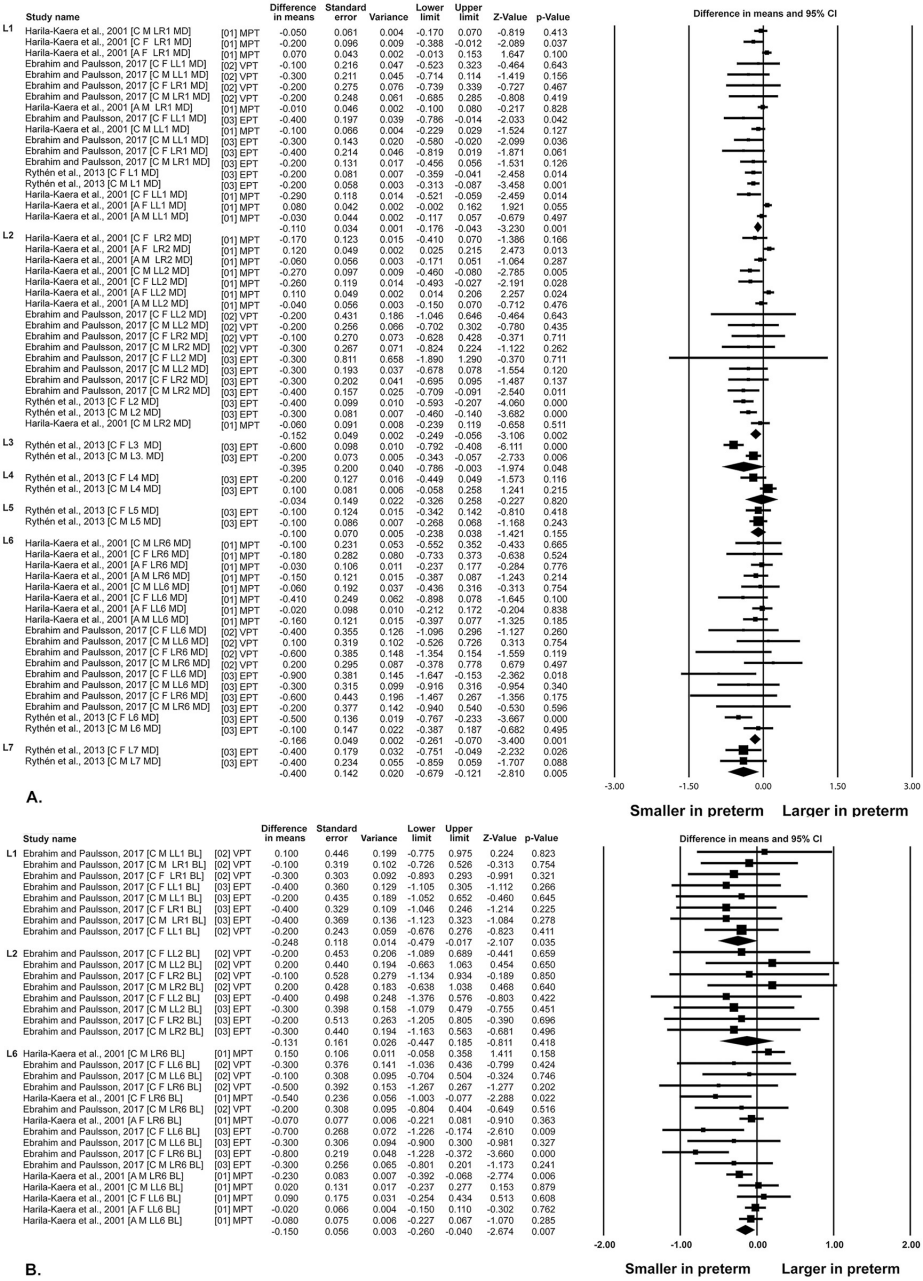
Differences in teeth dimension between preterm and control children. Mandibular teeth. A. Mesiodistal dimensions. B. Buccolingual dimensions. A: African American; BL: Buccolingual; C: Caucausian; CI: Confidence Interval; EPT; Extremely preterm; F: Females; LL: Mandibular left teeth; LL: Mandibular right teeth;M: Males; MD: Mesiodistal; MPT: Moderate preterm; VPT: Very preterm.

Overall, the mesiodistal and the buccolingual dimensions of the permanent teeth in both dental arches tended to be smaller in children born prematurely than full term children, with the EPT children groups showing the major differences (Figs 3 and 4).

#### Mesiodistal dimensions

Overall quantitative synthesis of the mesiodistal dimensions for each tooth across gestational age groups (Table 3), showed statistically significant differences for the maxillary central incisors [WMD: -0.150; 95% CI: -0.231 to -0.069], first molars [WMD: -0.174; 95% CI: -0.264 to -0.084] and second molars [WMD: -0.305; 95% CI: -0.585 to -0.024], as well as for mandibular central incisors [WMD: -0.177; 95% CI: -0.167 to -0.067], lateral incisors [WMD: -0.187; 95% CI: -0.256 to -0.118], canines [WMD: -0.395; 95% CI: -0.786 to -0.003], first molars [WMD:-0.136; 95% CI: -0.223 to -0.050] and second molars [WMD: -0.142; 95% CI: -0.679 to -0.121].

**Table 3:** Differences in permanent teeth dimensions between preterm and full-term children—quantitative synthesis statistics.

				Effect size and 95% confidence interval^§^										Effect size and 95% confidence interval^§^						
	Tooth	Groups	Datasets	WMD	SE	LL	UL	P-value	I^2^ (%)	Between groups	Tooth	Groups	Datasets	WMD	SE	LL	UL	P-value	I^2^ (%)	Between groups
**MD**	**U1**	MPT	8 (37)	-0.028	0.048	-0.122	0.067	0.0567	50.12	**0.000**	**L1**	MPT	8 (37)	-0.034	0.034	-0.101	0.033	0.326	65.4	**0.001**
		VPT	4 (39)	-0.396	0.195	-0.779	-0.013	0.043	0.00			VPT	4 (39)	-0.201	0.117	-0.430	0.028	0.085	0.00	
		EPT	6 (38, 39)	-0.527	0.090	-0.704	-0.351	**0.000**	22.37			EPT	6 (38, 39)	-0.224	0.041	-0.304	-0.144	**0.000**	0.00	
		**Overall**		-0.233	0.066	-0.363	-0.104	**0.000**	73.08			**Overall**		-0.110	0.034	-0.176	-0.043	**0.001**	64.79	
		***95% Prediction Interval***				-0.694	0.228					***95% Prediction Interval***				-0.334	0.114			
	**U2**	MPT	8 (37)	-0.028	0.070	-0.165	0.109	0.687	51.37	0.804	**L2**	MPT	8 (37)	-0.053	0.050	-0.150	0.045	0.288	74.78	**0.000**
		VPT	4 (39)	-0.122	0.152	-0.421	0.176	0.422	0.00			VPT	4 (39)	-0.200	0.144	-0.482	0.081	0.163	0.00	
		EPT	6 (38, 39)	-0.010	0.077	-0.161	0.141	0.899	0.00			EPT	6 (38, 39)	-0.341	0.054	-0.447	-0.236	**0.000**	0.00	
		**Overall**		-0.021	0.045	-0.109	0.066	0.631	17.80			**Overall**		-0.152	0.049	-0.249	-0.056	**0.002**	73.39	
		***95% Prediction Interval***				-0.210	0.168					***95% Prediction Interval***				-0.496	0.192			
	**U3**	EPT	2 [38]	-0.443	0.250	-0.933	0.047	0.076	93.03		**L3**	EPT	2 [38]	-0.395	0.200	-0.786	-0.003	**0.048**	90.62	
		**Overall**		-0.443	0.250	-0.933	0.047	0.076	93.03			**Overall**		-0.395	0.200	-0.786	-0.003	**0.048**	90.62	
	**U4**	EPT	2 [38]	-0.033	0.149	-0.325	0.259	0.823	76.04		**L4**	EPT	2 [38]	-0.034	0.149	-0.326	0.258	0.820	74.81	
		**Overall**		-0.033	0.149	-0.325	0.259	0.823	76.04			**Overall**		-0.034	0.149	-0.326	0.258	0.820	74.81	
	**U5**	EPT	2 [38]	-0.100	0.066	-0.229	0.029	0.127	0.00		**L5**	EPT	2 [38]	-0.100	0.070	-0.238	0.038	0.155	0.00	
		**Overall**		-0.100	0.066	-0.229	0.029	0.127	0.00			**Overall**		-0.100	0.070	-0.238	0.038	0.155	0.00	
	**U6**	MPT	8 (37)	-0.008	0.060	-0.126	0.109	0.888	60.38	**0.000**	**L6**	MPT	8 (37)	-0.095	0.050	-0.192	0.003	0.056	0.00	0.099
		VPT	4 (39)	-0.213	0.152	-0.512	0.086	0.163	0.00			VPT	4 (39)	-0.123	0.189	-0.494	0.247	0.514	21.67	
		EPT	6 (38, 39)	-0.471	0.082	-0.631	-0.312	**0.000**	15.31			EPT	6 (38, 39)	-0.363	0.114	-0.587	-0.139	**0.002**	25.34	
		**Overall**		-0.193	0.069	-0.329	-0.057	**0.005**	73.52			**Overall**		-0.166	0.049	-0.261	-0.070	**0.001**	15.67	
		***95% Prediction Interval***				-0.693	0.307					***95% Prediction Interval***				-0.360	0.028			
	**U7**	EPT	2 [38]	-0.305	0.143	-0.585	-0.024	**0.033**	39.54		**L7**	EPT	2 [38]	-0.400	0.142	-0.679	-0.121	**0.005**	0.00	
		**Overall**		-0.305	0.143	-0.585	-0.024	**0.033**	39.54			**Overall**		-0.400	0.142	-0.679	-0.121	**0.005**	0.00	
**BL**	**U1**	VPT	4 (39)	-0.202	0.241	-0.674	0.271	0.403	0.00	0.865	**L1**	VPT	4 (39)	-0.167	0.153	-0.467	0.133	0.274	0.00	0.411
		EPT	4 (39)	-0.254	0.186	-0.619	0.112	0.174	0.00			EPT	4 (39)	-0.364	0.184	-0.725	-0.004	**0.048**	0.00	
		**Overall**		-0.234	0.148	-0.523	0.055	0.112	0.00			**Overall**		-0.248	0.118	-0.479	-0.017	**0.035**	0.00	
	**U2**	VPT	4 (39)	0.227	0.226	-0.215	0.670	0.314	0.00	0.360	**L2**	VPT	4 (39)	0.042	0.229	-0.407	0.490	0.856	0.00	0.288
		EPT	4 (39)	-0.108	0.289	-0.673	0.458	0.709	0.00			EPT	4 (39)	-0.301	0.228	-0.747	0.145	0.186	0.00	
		**Overall**		0.100	0.178	-0.249	0.449	0.574	0.00			**Overall**		-0.131	0.161	-0.447	0.185	0.418	0.00	
	**U6**	MPT	8 (37)	-0.019	0.034	-0.085	0.048	0.580	0.00	**0.003**	**L6**	MPT	8 (37)	-0.060	0.050	-0.158	0.038	0.228	49.26	**0.002**
		VPT	4 (39)	-0.199	0.161	-0.515	0.118	0.218	0.00			VPT	4 (39)	-0.247	0.170	-0.579	0.086	0.147	0.00	
		EPT	4 (39)	-0.521	0.151	-0.817	-0.225	**0.001**	0.00			EPT	4 (39)	-0.561	0.133	-0.822	-0.299	**0.000**	7.20	
		**Overall**		-0.063	0.037	-0.136	0.011	0.093	12.80			**Overall**		-0.150	0.056	-0.260	-0.040	**0.007**	54.39	
		***95% Prediction Interval***				-0.205	0.079					***95% Prediction Interval***				-0.483	0.183			

BL: buccolingual; EPT; Extremely preterm (birth before completed 28 weeks of gestation); L: mandibular teeth; LL: Lower limit; MD: Mesiodistal; MPT: Moderate preterm (birth at completed 32 weeks of gestation but before completing 37 weeks); SE: Standard Error; U: Maxillary teeth; UL: Upper limit; VPT: Very preterm (birth at completed 28 weeks of gestation but before completing 32 weeks); WMD: Weighted Mean Difference

^**§**^Statistically significant differences in bold.

Comparisons per subgroups showed statistically significant differences for the EPT/control comparisons in the case of the maxillary central incisors, first and second molars, as well as mandibular central and lateral incisors, canines, first and second molars (Table 3). Comparisons between subgroups, showed statistically significant differences for the maxillary central incisors, first molars and mandibular central and lateral incisors (Table 3).

#### Buccolingual dimensions

Overall, quantitative synthesis of the buccolingual dimensions for each tooth across gestational age groups (Table 3), showed statistically significant differences for the mandibular central incisors [WMD: -0.248; 95% CI: -0.479 to -0.017] and first molars [WMD: -0.130; 95% CI: -0.218 to -0.042].

Comparisons per subgroups showed statistically significant differences for the EPT/control comparisons in the case of the maxillary first molars, as well as mandibular central incisors and first molars (Table 3). Comparisons between subgroups, showed statistically significant differences for the maxillary and mandibular first molars (Table 3).

#### Meta-regression and certainty of assessment

**Fig 5:**
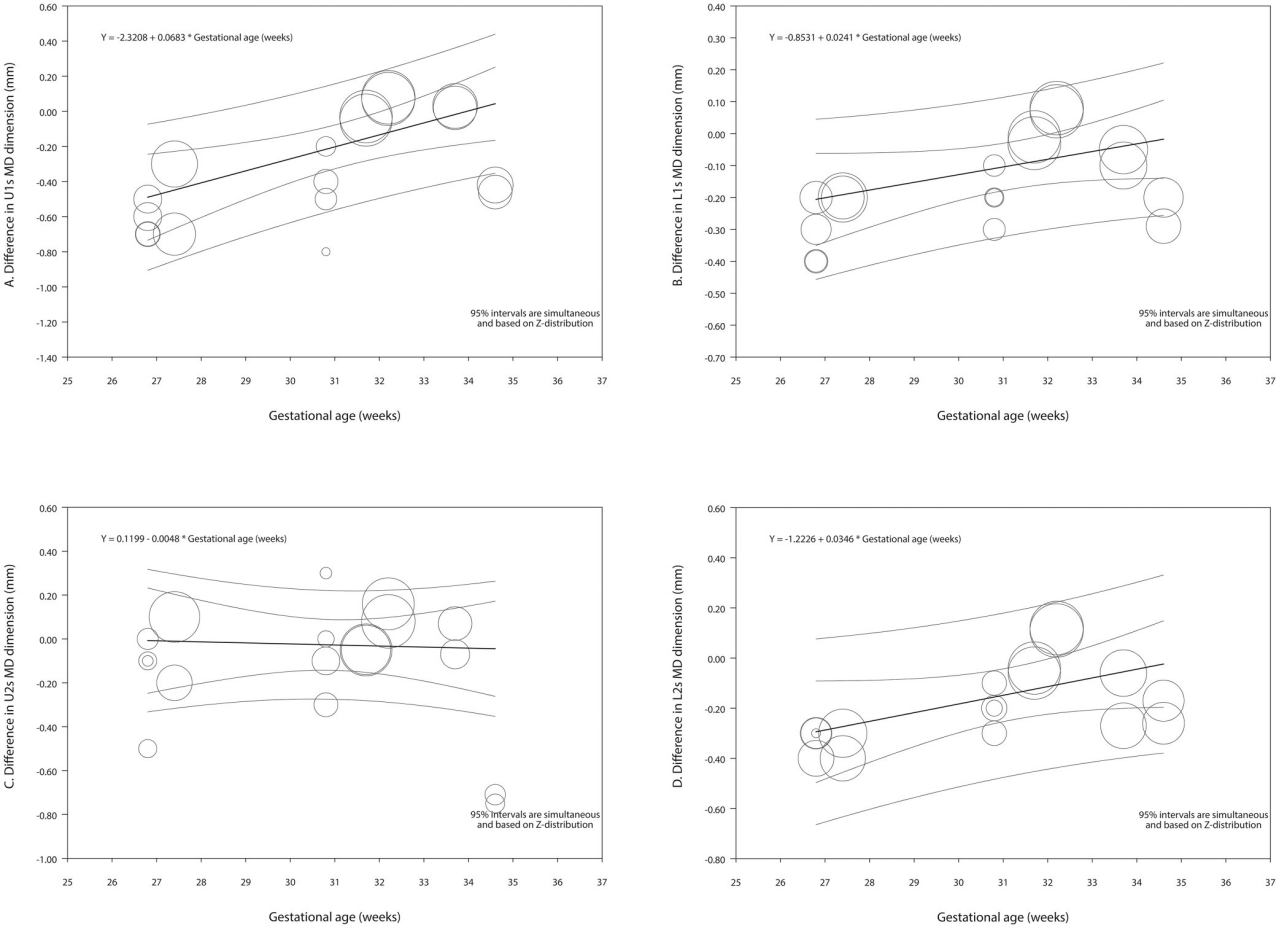
Regression scatterplots of the difference in teeth dimension between preterm and control children, on the predictor of gestational age (in weeks). A. Maxillary central incisor—Mesiodistal dimension. B. Mandibular central incisor—Mesiodistal dimension. C. Maxillary lateral incisor—Mesiodistal dimension. D. Mandibular lateral incisor—Mesiodistal dimension. Confidence intervals and prediction intervals are displayed (inner and outer lines around the regression line). MD: Mesiodistal; L: Lower dental arch; U: Upper dental arch.

**Fig 6:**
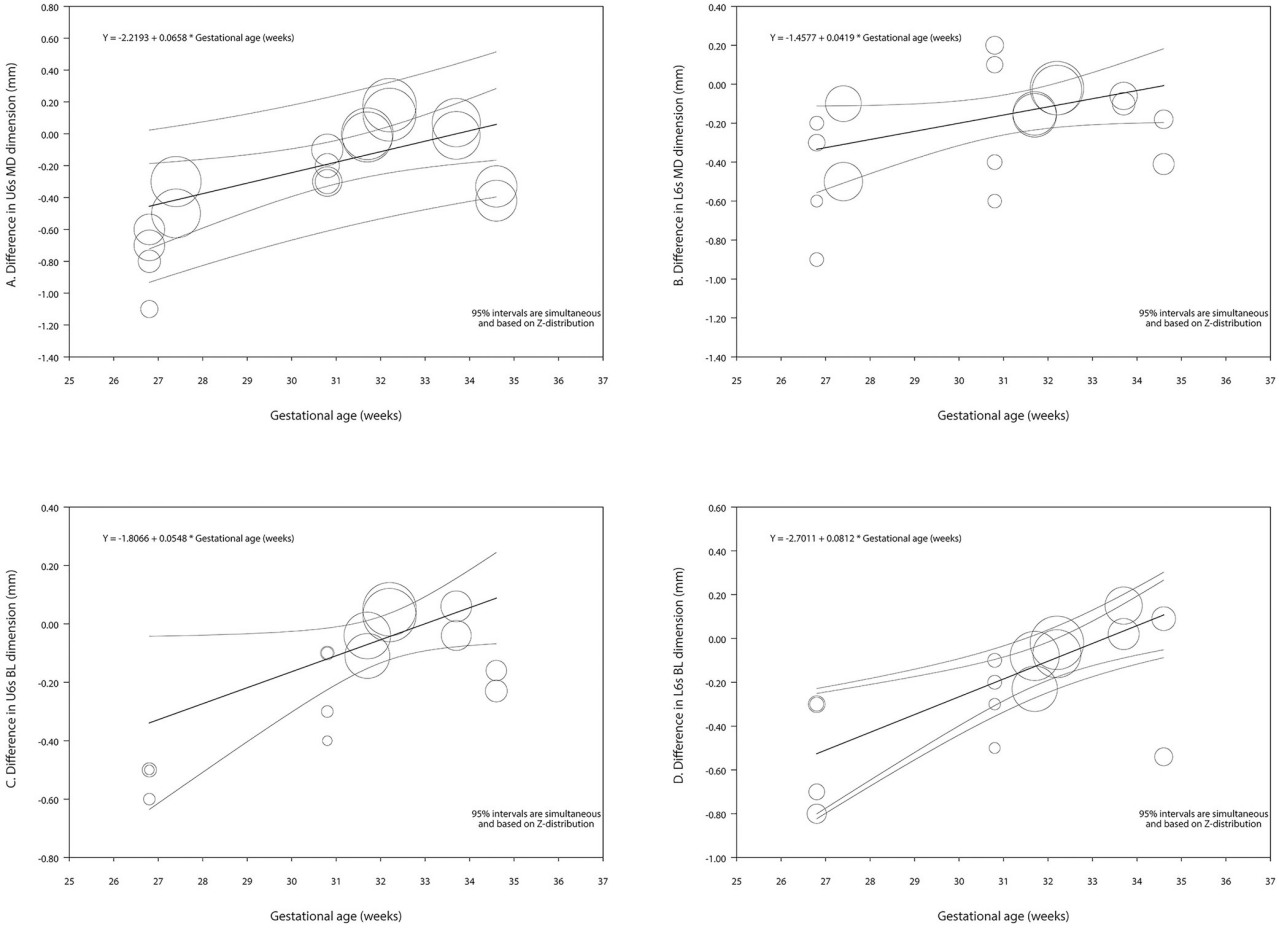
Regression scatterplots of the difference in teeth dimension between preterm and control children, on the predictor of gestational age (in weeks). A. Maxillary first molar—Mesiodistal dimension. B. Mandibular first molar—Mesiodistal dimension. C. Maxillary first molar—Buccolingual dimension. D. Mandibular first molar—Buccolingual dimension. Confidence intervals and prediction intervals are displayed (inner and outer lines around the regression line). BL: Buccolingual; MD: Mesiodistal; L: Lower dental arch; U: Upper dental arch.

In the series of exploratory meta-regressions for the differences in the mesiodistal dimensions that included the intercept and the gestation age in weeks as predictor, with the exception of the maxillary lateral incisor, the coefficient for the gestation age had a range up to 0.068 for the maxillary central incisor, i.e., the maxillary central incisor became smaller in premature the children by 0.27 mm for every month of prematurity compared those born at full term (Table 4, Figs 5 and 6). In the meta-regressions for the differences in the buccolingual dimensions, the coefficient for the gestation age was 0.054 and 0.082 for the maxillary and mandibular first molar respectively. In other words, these teeth became smaller in premature the children by 0.22 mm and 0.32 mm for every month of prematurity compared those born at full term (Table 4, Figs 5 and 6).

**Table 4:** Meta-regression for the effect on the differences in permanent teeth dimensions between preterm and full-term children. Main results and statistics for the univariate regression models.

		Main results^1^				Test of the model			Goodness of fit					Main results^1^				Test of the model			Goodness of fit			
	Tooth	Coefficient	SE	LL	UL	Q	df	p-value	Q	df	p-value	R^2^ analog	Tooth	Coefficient	SE	LL	UL	Q	df	p-value	Q	df	p-value	R^2^ analog
**MD**		**Gestational age (in weeks)**												**Gestational age (in weeks)**										
	**U1**	0.068	0.019	0.029	0.107	11.91	1	**0.000**	35.24	16	0.003	0.56	**L1**	0.024	0.011	0.001	0.046	4.34	1	**0.037**	36.20	16	0.002	0.31
	**U2**	-0.004	0.020	-0.045	0.035	0.05	1	0.817	20.65	16	0.192	0.00	**L2**	0.034	0.016	0.0028	0.066	4.56	1	**0.032**	45.68	16	0.00	0.33
	**U6**	0.065	0.021	0.023	0.107	9.42	1	**0.002**	38.31	16	0.01	0.49	**L6**	0.041	0.018	0.005	0.078	5.03	1	**0.025**	15.14	16	0.514	1.00
	**Birth weight (in g)**												**Birth weight (in g)**											
	**U1**	0.0002	0.0003	-0.0004	0.0008	0.39	1	0.532	6.81	8	0.557	0.00	**L1**	0.0001	0.0002	-0.0003	0.0004	0.10	1	0.752	2.53	8	0.965	0.00
	**U2**	-0.0002	0.0003	-0.0007	0.0004	0.33	1	0.564	5.68	8	0.683	0.00	**L2**	0.0002	0.0002	-0.0002	0.0007	0.83	1	0.361	1.13	8	0.997	0.00
	**U6**	0.0004	0.0003	-0.0001	0.0009	2.49	1	0.114	5.74	8	0.675	0.00	**L6**	0.0004	0.0003	-0.0003	0.0010	1.42	1	0.233	10.37	8	0.240	0.15
		**Racial background** ^2^												**Racial background** ^2^										
	**U1**	-0.371	0.099	-0.565	-0.176	13.98	1	**0.000**	32.54	16	0.008	0.60	**L1**	-0.199	0.034	-0.267	0.131	32.91	1	**0.000**	15.38	16	0.497	1.00
	**U2**	-0.128	0.087	-0.299	0.042	2.17	1	0.140	18.57	16	0.291	0.21	**L2**	-0.295	0.051	-0.395	-0.195	33.65	1	**0.000**	18.85	16	0.176	0.93
	**U6**	-0.372	0.104	-0.577	0.168	12.72	1	**0.000**	31.90	16	0.010	0.62	**L6**	-0.177	0.084	-0.343	0.011	4.39	1	**0.036**	20.16	16	0.469	1.00
		**Gender** ^3^												**Gender** ^3^										
	**U1**	-0.126	0.140	-0.402	0.149	0.81	1	0.367	63.16	16	0.000	0.00	**L1**	0.005	0.068	-0.129	0.140	0.01	1	0.934	43.90	16	0.000	0.00
	**U2**	-0.013	0.096	-0.201	0.174	0.02	1	0.889	20.52	16	0.197	0.00	**L2**	0.074	0.095	-0.112	0.260	0.61	1	0.436	52.22	16	0.000	0.10
	**U6**	-0.062	0.145	-0.347	0.222	0.19	1	0.667	63.98	16	0.000	0.00	**L6**	-0.118	0.099	-0.313	0.077	1.40	1	0.236	19.38	16	0.249	0.00
**BL**		**Gestational age (in weeks)**												**Gestational age (in weeks)**										
	**U1**	0.015	0.074	-0.130	0.160	0.04	1	0.839	0.35	6	0.999	0.00	**L1**	0.049	0.059	-0.068	0.166	0.68	1	0.410	0.79	6	0.992	0.00
	**U2**	0.083	0.091	-0.095	0.264	0.84	1	0.360	0.56	6	0.997	0.00	**L2**	0.085	0.080	-0.072	0.243	1.13	1	0.288	0.70	6	0.994	0.00
	**U6**	0.054	0.022	0.011	0.097	6.18	1	**0.012**	11.02	14	0.684	1.00	**L6**	0.082	0.020	0.040	0.121	15.33	1	**0.000**	15.79	14	0.326	0.90
	**Birth weight (in g)**												**Birth weight (in g)**											
	**U1**	0.0001	0.0004	-0.0007	0.0009	0.04	1	0.839	0.35	6	0.999	0.00	**L1**	0.0003	0.0003	-0.0004	0.001	0.68	1	0.410	0.79	6	0.992	0.00
	**U2**	0.0005	0.0005	-0.0005	0.0015	0.84	1	0.360	0.56	6	0.997	0.00	**L2**	0.0005	0.0005	-0.0004	0.0014	1.13	1	0.288	0.70	6	0.994	0.00
	**U6**	0.0005	0.0003	-0.0002	0.0011	2.12	1	0.144	0.64	6	0.995	0.00	**L6**	0.0005	0.0003	-0.0001	0.0010	2.22	1	0.136	3.92	6	0.687	0.00
		**Racial background** ^2^												**Racial background** ^2^										
	**U6**	-0.143	0.071	-0.284	-0.003	4.01	1	**0.045**	13.19	14	0.511	1.00	**L6**	-0.106	0.117	-0.336	0.123	0.82	1	0.364	32.69	14	0.003	0.00
		**Gender** ^3^												**Gender** ^3^										
	**U1**	0.051	0.186	-0.545	0.649	0.03	1	0.865	0.36	6	0.999	0.00	**L1**	-0.137	0.242	-0.612	0.338	0.32	1	0.572	1.14	6	0.979	0.00
	**U2**	-0.036	0.356	-0.734	0.662	0.01	1	0.919	1.39	6	0.966	0.00	**L2**	-0.167	0.326	-0.807	0.472	0.26	1	0.607	1.57	6	0.955	0.00
	**U6**	0.034	0.077	-0.118	0.187	0.20	1	0.656	16.46	14	0.286	0.00	**L6**	-0.145	0.120	-0.380	0.090	1.45	1	0.227	32.73	14	0.003	0.00

L: Mandibular teeth; LL: Lower limit; SE: Standard Error; U: Maxillary teeth; UL: Upper limit.

^1^Random effects (Method of Moments), Z-Distribution, statistically significant effects in bold.

^2^Reference group: African American.

^3^Reference group: Male.

In the series of exploratory meta-regressions for the differences in the mesiodistal dimensions that included the intercept and the racial background as predictor, except for the maxillary lateral incisor, it was observed, that the amount the teeth would be smaller in the premature children compared to those born full term, was greater in Caucasians than African Americans. For the buccolingual dimensions the same was observed only for the maxillary first molar. No statistically significant results were noted for the models including the birth weight or the gender as predictors (Table 4).

In the multivariate regression models incorporating the intercept and the statistically significant variables of gestational age and racial background as predictors, statistically significant results were noted for the maxillary central incisors and first molars, as well as mandibular central incisor, lateral incisor and first molar in the mesiodistal dimension. In the buccolingual dimension, statistically significant results were noted for both maxillary and mandibular first molars. The R^2^ analogue ranged from 0.84 to 0.90, i.e., gestational age and racial background explain almost all of the variance in true effects (Table 5).

**Table 5:** Meta-regression for the effect on the differences in permanent teeth dimensions between preterm and full-term children. Statistics for the multivariate regression models.

		Test of the model^1^			Goodness of fit			
Dimension	Tooth	Q	df	p-value	Q	df	p-value	R^2^ analog
**MD**	**U1**	33.74	2	**0.000**	18.84	15	0.220	0.90
	**U6**	31.87	2	**0.000**	18.83	15	0.001	0.90
	**L1**	36.93	2	**0.000**	11.35	15	0.727	1.00
	**L2**	50.82	2	**0.000**	13.09	15	0.595	1.00
	**L6**	6.46	2	**0.039**	13.70	15	0.548	1.00
**BL**	**U6**	11.58	2	**0.003**	5.63	13	0.958	1.00
	**L6**	15.17	2	**0.000**	15.46	13	0.179	0.84

^1^[Gestational age and racial group (Reference: African American)], Random effects (Method of Moments), Z-Distribution, statistically significant effects in bold.

Regarding the differences in the permanent teeth dimensions between preterm and full-term children, the quality of available evidence was rated at best as moderate (S3 Table) [28, 31].

## Discussion

The regular cycle of permanent teeth formation process may be disrupted in preterm infants [9], with potential discrepancies in size and subsequent occlusal disturbances [10]. The results of the present review suggest that premature birth could potentially be associated with reduced tooth-crown dimensions in some permanent teeth. Moreover, this difference seemed to become more pronounced with decreasing gestational age. Although the results from these observational studies should be approached with some caution until more information becomes available, the clinician should not overlook the possible clinical implications.

Overall, the mesiodistal and the buccolingual dimensions of the permanent teeth in both dental arches tended to be smaller in children born prematurely than full term children, with the EPT children groups showing more pronounced differences. Quantitative syntheses of the mesiodistal dimensions for each tooth across different prematurity groups showed statistically significant differences for the centrals, first and second molars in the maxilla, as well as centrals, laterals, canines, first and second molars in the mandible.

Corroborating were the results of the univariate meta-regressions with the gestation age as predictor, which showed statistically significant associations for the mesiodistal dimensions of all investigated teeth, except for the maxillary lateral, as well as the buccolingual dimensions for the maxillary and mandibular first molars. Moreover, analyses per subgroups of prematurity showed statistically significant differences between the EPT and control groups for several comparisons as well [Mesiodistal dimensions: maxillary centrals, first and second molars; mandibular centrals laterals, canines, first and second molars] [Buccolingual dimensions: maxillary first molars; mandibular centrals, first molars].

Maxillary and mandibular centrals, canines and first molars, as well as mandibular laterals form early and develop at a period that can be potentially more affected from a premature birth [41]. The maxillary laterals did not show significant differences, potentially because it is the most dimensionally variable tooth in the dentition [10] and develops at later period [41] that may be not so influenced by the immediate effect of premature birth. Moreover, they might be commonly unavailable for measuring because of congenital absence or because in delays in eruption in children in the mixed dentition [39].

The second molars also develop at a later stage [41]. In these cases, the disturbance in crown formation could potentially be attributed to space conditions at the dental arches at critical periods for their development [42]. Premature birth has been reported to result in alterations in craniofacial morphology in young children, that were found to have significantly shorter anterior cranial bases, shorter maxillary lengths and a less convex skeletal profiles compared to full-term children, especially those born extremely pre-term [43]. The length of the mandibular arch might not be as developed, as well [43]. Although the formation of the second molars starts at 2–3 years of age [41], delays in dental maturation can be expected [42]. So, these teeth might develop in an area when the dental arches are not as developed as in full term children because of prematurity, and since they develop late might not have the chance to benefit from the period of catch-up growth that can be observed in prematurely born individuals [42].

In two out of the three included studies, the buccolingual dimensions were measured as well, but for fewer teeth. Statistically significant differences were detected for mandibular centrals and maxillary/mandibular first molars. Dimensional variations as well as difficulties in identifying reference points for measurements in the buccolingual direction could be implicated. Moreover, the variation in tooth shape might result in problems when applying some of the measurement definitions [44, 45].

In the cases of the quantitative syntheses, for each tooth dimensions, across gestational age groups, it was possible to calculate prediction intervals. Prediction intervals facilitate the assessment of heterogeneity for each combined comparison and constitute the intervals expected to include the true intervention effects in future studies, or exposure effects in this specific case [15]. In our case the prediction intervals were wide enough to suggest that future studies could detect no effect on tooth size from the preterm birth across gestational age groups. The possibility of no effect as suggested by the prediction intervals could possibly be related to the inclusion of the Harila-Kaera et al. study [37] which assessed children born in the 1960s, when care was not as advanced as today and extremely preterm children might have less chances for survival that they exhibit nowadays [40]. Indeed, this was reflected in the higher gestational ages of the children included in the preterm groups in the abovementioned paper, compared to the other two. Furthermore, this effect could become even more pronounced as the Harila-Kaera et al. [37] study assessed significantly more individuals than the other included papers, thus carrying more weight in the corresponding analyses.

Investigation of the associated heterogeneity included a series of exploratory meta-regressions. The multivariate regression models including the gestational age and racial background as predictors, showed statistically significant associations with R^2^ analogue ranging from 0.84 to 0.90, i.e., gestational age and racial background explain almost all the variance in true effects. These results add to the body of evidence that the regular cycle of permanent teeth formation process may be disrupted in preterm infants [9].

The meta-regressions including the racial background as predictor, showed that the difference between preterm and full-term children would be greater in Caucasians. That was observed for all studied maxillary teeth mesiodistal dimensions, except for the maxillary lateral incisor, and the maxillary first molar buccolingual dimension. The differences observed between different ethnic groups are thought to reflect differences in the relative contributions of genetics and environmental influences on dental development [46].

Meta-regression showed no statistically significant results for the models including the birth weight or the gender as predictors. Low birth weight is directly related to low gestational ages and birth prematurity [6], and it has been correlated with smaller tooth size [33, 47]. Moreover, there is a possible relationship between overall body and head growth and the process of final determination of dental dimensions. This may be consistent with increased cellular activity in the developing tooth germs during the catch-up growth period causing quantitative changes in the teeth size which are at critical stage of development at that time [48]. However, low birth weight can be also the result of restricted intrauterine growth. Thus, birth weight might not accurately describe the degree of immaturity of the neonate [6].

Regarding the influence of gender, it has been suggested that differences exist in the development of the dentition. Boys generally have larger teeth than girls [49]. The genes affecting tooth crown size are situated on both the X and Y chromosome. The influence of the Y chromosome is different from the X chromosome. The X chromosome seems to promote enamel formation, whereas the Y-chromosome to influence the formation of both enamel and dentine [50].

Other factors that might influence tooth size determination include poor maternal health during pregnancy (hypothyroidism, diabetes, hypertension, etc.) [47] and maternal smoking during pregnancy [51]. Consequently, it is possible that the obtained results have arisen from confounding by other parameters apart from those explored in this manuscript. However, the available variables in the dataset limited further exploration.

Even though overall evidence assessment provides the clinician with a variable perspective on the strength of the relevant findings, it could constitute good practice for the clinician to be able to identify such patients and consider the possible implications. The dimensional discrepancies found in this study for some of the permanent teeth might be useful for the orthodontists while predicting orthodontic treatment needs, planning for future space requirements in the mixed dentition stage, as well as in the comprehensive orthodontic treatment phase for prematurely born children, especially the extremely preterm group. Preterm children have been found to have spacing [43], which could be connected to the smaller tooth dimensions observed in the context of the present study. Spacing and smaller teeth could also be aesthetically problematic. Especially anterior spacing might impact Oral Health Related Quality of Life negatively and increase self-consciousness about one’s appearance [52–54]. Moreover, when permanent teeth are not matched for size, normal occlusion might be difficult to achieve [10]. Other malocclusion traits reported for preterm children include posterior crossbite [55] and increased overbite [43, 56].

### Strengths and limitations

The fact that this study followed well-established guidelines counts as strength for the present review. A comprehensive literature search with no restrictions until March 2021 was conducted, thus including every available relevant study. All procedures were performed in duplicate in an effort to make the possibility of bias less and disagreements were resolved by discussion. Also, in order to address eventual heterogeneity in exploratory data synthesis we employed the model of random effects and we used meta-regression to explore whether gestational age, birth weight, racial background and gender modified the results [15].

Another strength of the present study is relevant to the definition of prematurity. We followed the World Health Organization, which follows the criterium of the gestational age; thus, making the possibility of consideration in the preterm groups of full-term children with low birthweight smaller (i.e., inclusion of infants that are mature but small for their gestational age). Moreover, the potential confounding effect of the racial differences was minimized for each sample, since populations with distinctive racial backgrounds were studied in the included studies.

Limitations to the present study surfaced mainly from nature and the characteristics of the included papers and the data retrieved during the review process. The fact that not many studies and population datasets could be located for some comparisons renders quantitative assessments indicative and exploratory until additional research becomes available. Nevertheless, alternative summaries can be less transparent and potentially less valid [57] and even information from two studies can be synthesized as long as pooling is meaningful [58].

The Newcastle-Ottawa scale assessments identified issues regarding the selection and comparability domains. Although important confounders were considered, other genetic or epigenetic influences were not measured or not controlled for, thus could not be explored in the context of the present dataset. It is important to note that the Harila-Kaera et al. [37] population might not reflect current preterm children since medical care has significantly advanced and has resulted in increased survival rates of infants of very small gestational age. Also, both Harila-Kaera et al. [37] and Rythén et al. [38] included premature children, irrespective of health status and did not exclude infants with syndromes or with neuromuscular disorders, like Ebrahim and Paulsson [39]. Notwithstanding the limited number of retrieved studies and the associated limitations, the present review offers valuable insights into the relationship of premature birth to tooth dimensions.

### Recommendations for future research

Advances of modern medicine have contributed to the increased survival rates of preterm children, especially those born at small gestational ages. Further well-designed observational studies are advised in order to fully investigate the effect of preterm birth not only on tooth size but only on other parameters related to oral health.

## Conclusions

Within the limitations of the present study, premature birth could potentially be associated with reduced tooth-crown dimensions in some permanent teeth, at least in children born extremely preterm. Although the results from these observational studies should be approached with some caution until more information becomes available, the clinician should not overlook the possible clinical implications in terms of diagnosis and treatment planning.

## Supporting information

S1 Table Eligibility criteria. (DOCX)

S2 Table Strategy for database search [February 17^th^, 2021]. (DOCX)

S3 Table Quality of available evidence. (DOCX)

S1 Checklist PRISMA 2020 checklist. (DOCX)
