# Supplementary material for: What is the effect of preterm birth on permanent tooth crown dimensions? A systematic review and meta-analysis
Source: PLoS One. 2021 Nov 5;16(11):e0259293. doi: 10.1371/journal.pone.0259293 (PMC8570496; doi:10.1371/journal.pone.0259293)
Supplement: S1 Table — (DOCX) [file pone.0259293.s001.docx]

**Supplementary Table 1.** Eligibility criteria for the present systematic review.

| **Domain** | **Inclusion criteria** | **Exclusion criteria** |
| --- | --- | --- |
| **Population** | - Individuals of any age, gender and racial background with permanent teeth (mixed or permanent dentition) | - Individuals with primary dentition |
| **Exposure** | - Preterm birth as per WHO criteria (verified by medical/hospital records) | - Gestational age at birth not verified by medical/hospital records |
| **Comparator** | - Full term birth (verified by medical/hospital records) | - Gestational age at birth not verified by medical/hospital records |
| **Outcomes** | - Mesiodistal and/or buccolingual dimensions of permanent teeth (along with measurements of dispersion). Where needed Standard Deviations were estimated following Wan et al., (2014). | - Not quantified measurements and qualitative assessments; absence of measures of central tendency and dispersion. |

Wan X, Wang W, Liu J, Tong T. Estimating the sample mean and standard deviation from the sample size, median, range and/or interquartile range. BMC Med Res Methodol 2014 Dec 19;14:135.
