# Supplementary material for: What is the effect of preterm birth on permanent tooth crown dimensions? A systematic review and meta-analysis
Source: PLoS One. 2021 Nov 5;16(11):e0259293. doi: 10.1371/journal.pone.0259293 (PMC8570496; doi:10.1371/journal.pone.0259293)
Supplement: S2 Table — (DOCX) [file pone.0259293.s002.docx]

**Supplementary Table 2.** Strategy for database search (up to March 14^th^, 2021).

| **Database** | **Search strategy** | **Hits** |
| --- | --- | --- |
| **Medline via PubMed** | (((“very preterm” OR “extreme preterm” OR premature OR preterm) AND infant) OR ((“very preterm” OR “extreme preterm” OR premature OR preterm) AND birth) OR prematurity) AND (“tooth morphology” OR “teeth morphology” OR “arch morphology” OR “arches morphology” OR “palate morphology” OR “palatal morphology” OR “cross bite” OR “cross-bite” OR “molar relation” OR “molars relation” OR “incisor relation” OR “incisors relation” OR “incisal relation” OR overjet OR overbite OR “open bite” OR “deep bite” OR ((intermolar OR inter-molar OR intercanine OR inter-canine OR interpremolar OR inter-premolar) AND width) OR “arch length” OR “arch depth” OR mesiodistal OR “mesio-distal” OR buccolingual OR “bucco-lingual” OR buccopalatal OR “bucco-palatal” OR “tooth maturity” OR “tooth maturation” OR “tooth eruption” OR “teeth maturity” OR “teeth maturation” OR “teeth eruption” OR “tooth abnormalities” OR “teeth abnormality”) | **426** |
| **Cochrane Central Register of Controlled Trials** | (((“very preterm” OR “extreme preterm” OR premature OR preterm) AND infant) OR ((“very preterm” OR “extreme preterm” OR premature OR preterm) AND birth) OR prematurity) AND (“tooth morphology” OR “teeth morphology” OR “arch morphology” OR “arches morphology” OR “palate morphology” OR “palatal morphology” OR “cross bite” OR “cross-bite” OR “molar relation” OR “molars relation” OR “incisor relation” OR “incisors relation” OR “incisal relation” OR overjet OR overbite OR “open bite” OR “deep bite” OR ((intermolar OR inter-molar OR intercanine OR inter-canine OR interpremolar OR inter-premolar) AND width) OR “arch length” OR “arch depth” OR mesiodistal OR “mesio-distal” OR buccolingual OR “bucco-lingual” OR buccopalatal OR “bucco-palatal” OR “tooth maturity” OR “tooth maturation” OR “tooth eruption” OR “teeth maturity” OR “teeth maturation” OR “teeth eruption” OR “tooth abnormalities” OR “teeth abnormality”) in Record Title OR (((“very preterm” OR “extreme preterm” OR premature OR preterm) AND infant) OR ((“very preterm” OR “extreme preterm” OR premature OR preterm) AND birth) OR prematurity) AND (“tooth morphology” OR “teeth morphology” OR “arch morphology” OR “arches morphology” OR “palate morphology” OR “palatal morphology” OR “cross bite” OR “cross-bite” OR “molar relation” OR “molars relation” OR “incisor relation” OR “incisors relation” OR “incisal relation” OR overjet OR overbite OR “open bite” OR “deep bite” OR ((intermolar OR inter-molar OR intercanine OR inter-canine OR interpremolar OR inter-premolar) AND width) OR “arch length” OR “arch depth” OR mesiodistal OR “mesio-distal” OR buccolingual OR “bucco-lingual” OR buccopalatal OR “bucco-palatal” OR “tooth maturity” OR “tooth maturation” OR “tooth eruption” OR “teeth maturity” OR “teeth maturation” OR “teeth eruption” OR “tooth abnormalities” OR “teeth abnormality”) in Abstract - (Word variations have been searched) | **0** |
| **Cochrane Database of Systematic Review** | (((“very preterm” OR “extreme preterm” OR premature OR preterm) AND infant) OR ((“very preterm” OR “extreme preterm” OR premature OR preterm) AND birth) OR prematurity) AND (“tooth morphology” OR “teeth morphology” OR “arch morphology” OR “arches morphology” OR “palate morphology” OR “palatal morphology” OR “cross bite” OR “cross-bite” OR “molar relation” OR “molars relation” OR “incisor relation” OR “incisors relation” OR “incisal relation” OR overjet OR overbite OR “open bite” OR “deep bite” OR ((intermolar OR inter-molar OR intercanine OR inter-canine OR interpremolar OR inter-premolar) AND width) OR “arch length” OR “arch depth” OR mesiodistal OR “mesio-distal” OR buccolingual OR “bucco-lingual” OR buccopalatal OR “bucco-palatal” OR “tooth maturity” OR “tooth maturation” OR “tooth eruption” OR “teeth maturity” OR “teeth maturation” OR “teeth eruption” OR “tooth abnormalities” OR “teeth abnormality”) in Record Title OR (((“very preterm” OR “extreme preterm” OR premature OR preterm) AND infant) OR ((“very preterm” OR “extreme preterm” OR premature OR preterm) AND birth) OR prematurity) AND (“tooth morphology” OR “teeth morphology” OR “arch morphology” OR “arches morphology” OR “palate morphology” OR “palatal morphology” OR “cross bite” OR “cross-bite” OR “molar relation” OR “molars relation” OR “incisor relation” OR “incisors relation” OR “incisal relation” OR overjet OR overbite OR “open bite” OR “deep bite” OR ((intermolar OR inter-molar OR intercanine OR inter-canine OR interpremolar OR inter-premolar) AND width) OR “arch length” OR “arch depth” OR mesiodistal OR “mesio-distal” OR buccolingual OR “bucco-lingual” OR buccopalatal OR “bucco-palatal” OR “tooth maturity” OR “tooth maturation” OR “tooth eruption” OR “teeth maturity” OR “teeth maturation” OR “teeth eruption” OR “tooth abnormalities” OR “teeth abnormality”) in Abstract - (Word variations have been searched) | **0** |
| **Scopus** | TITLE-ABS-KEY (((("very preterm" OR "extreme preterm" OR premature OR preterm) AND infant) OR (("very preterm" OR "extreme preterm" OR premature OR preterm) AND birth) OR prematurity) AND ("tooth morphology" OR "teeth morphology" OR "arch morphology" OR "arches morphology" OR "palate morphology" OR "palatal morphology" OR "cross bite" OR "cross-bite" OR "molar relation" OR "molars relation" OR "incisor relation" OR "incisors relation" OR "incisal relation" OR overjet OR overbite OR "open bite" OR "deep bite" OR ((intermolar OR inter-molar OR intercanine OR inter-canine OR interpremolar OR inter-premolar) AND width) OR "arch length" OR "arch depth" OR mesiodistal OR "mesio-distal" OR buccolingual OR "bucco-lingual" OR buccopalatal OR "bucco-palatal" OR "tooth maturity" OR "tooth maturation" OR "tooth eruption" OR "teeth maturity" OR "teeth maturation" OR "teeth eruption" OR "tooth abnormalities" OR "teeth abnormality")) | **158** |
| **Web of Knowledge ™** | TOPIC: ((((“very preterm” OR “extreme preterm” OR premature OR preterm) AND infant) OR ((“very preterm” OR “extreme preterm” OR premature OR preterm) AND birth) OR prematurity) AND (“tooth morphology” OR “teeth morphology” OR “arch morphology” OR “arches morphology” OR “palate morphology” OR “palatal morphology” OR “cross bite” OR “cross-bite” OR “molar relation” OR “molars relation” OR “incisor relation” OR “incisors relation” OR “incisal relation” OR overjet OR overbite OR “open bite” OR “deep bite” OR ((intermolar OR inter-molar OR intercanine OR inter-canine OR interpremolar OR inter-premolar) AND width) OR “arch length” OR “arch depth” OR mesiodistal OR “mesio-distal” OR buccolingual OR “bucco-lingual” OR buccopalatal OR “bucco-palatal” OR “tooth maturity” OR “tooth maturation” OR “tooth eruption” OR “teeth maturity” OR “teeth maturation” OR “teeth eruption” OR “tooth abnormalities” OR “teeth abnormality”))  Timespan: All years. Databases:WOS, KJD, RSCI, SCIELO, ZOOREC.  Search language=Auto | **118** |
| **ProQuest Dissertations and Theses Global** | ti((((“very preterm” OR “extreme preterm” OR premature OR preterm) AND infant) OR ((“very preterm” OR “extreme preterm” OR premature OR preterm) AND birth) OR prematurity) AND (“tooth morphology” OR “teeth morphology” OR “arch morphology” OR “arches morphology” OR “palate morphology” OR “palatal morphology” OR “cross bite” OR “cross-bite” OR “molar relation” OR “molars relation” OR “incisor relation” OR “incisors relation” OR “incisal relation” OR overjet OR overbite OR “open bite” OR “deep bite” OR ((intermolar OR inter-molar OR intercanine OR inter-canine OR interpremolar OR inter-premolar) AND width) OR “arch length” OR “arch depth” OR mesiodistal OR “mesio-distal” OR buccolingual OR “bucco-lingual” OR buccopalatal OR “bucco-palatal” OR “tooth maturity” OR “tooth maturation” OR “tooth eruption” OR “teeth maturity” OR “teeth maturation” OR “teeth eruption” OR “tooth abnormalities” OR “teeth abnormality”)) OR ab((((“very preterm” OR “extreme preterm” OR premature OR preterm) AND infant) OR ((“very preterm” OR “extreme preterm” OR premature OR preterm) AND birth) OR prematurity) AND (“tooth morphology” OR “teeth morphology” OR “arch morphology” OR “arches morphology” OR “palate morphology” OR “palatal morphology” OR “cross bite” OR “cross-bite” OR “molar relation” OR “molars relation” OR “incisor relation” OR “incisors relation” OR “incisal relation” OR overjet OR overbite OR “open bite” OR “deep bite” OR ((intermolar OR inter-molar OR intercanine OR inter-canine OR interpremolar OR inter-premolar) AND width) OR “arch length” OR “arch depth” OR mesiodistal OR “mesio-distal” OR buccolingual OR “bucco-lingual” OR buccopalatal OR “bucco-palatal” OR “tooth maturity” OR “tooth maturation” OR “tooth eruption” OR “teeth maturity” OR “teeth maturation” OR “teeth eruption” OR “tooth abnormalities” OR “teeth abnormality”)) | **1** |

|  |  |
| --- | --- |
|  |  |
|  |  |
|  |  |
|  |  |
