# Supplementary material for: What is the effect of preterm birth on permanent tooth crown dimensions? A systematic review and meta-analysis
Source: PLoS One. 2021 Nov 5;16(11):e0259293. doi: 10.1371/journal.pone.0259293 (PMC8570496; doi:10.1371/journal.pone.0259293)
Supplement: S3 Table — (DOCX) [file pone.0259293.s003.docx]

**Supplementary Table 3.** Quality of available evidence.

|  | - **Quality assessment** | | | | | | - **Effect size** | - **Quality** | |
| --- | --- | --- | --- | --- | --- | --- | --- | --- | --- |
| - **Teeth** | - **Studies** | - **Risk of bias** | - **Inconsistency** | - **Indirectness** | - **Imprecision** | - **Other** | - **WMD and 95% CI** |  | |
|  | **Differences in permanent teeth dimensions between preterm and full-term children - Mesiodistal dimensions** | | | | | | | | |
| - **U1** | 3 | - Serious^1^ | - Serious^2^ | - Not serious | - Not serious | - None | - 0.150 smaller in the PT group [from -0.231 to -0.069; p=0.000] | - ⨁⨁⨁◯ | **MODERATE^3^** |
| - **U2** | 3 | - Serious^1^ | - Serious^2^ | - Not serious | - Not serious | - None | - 0.030 smaller in the PT group [from -0.127 to 0.066; p=0.534] | - ⨁⨁◯◯ | - **LOW** |
| - **U3** | 1 | - Serious^1^ | - Serious^2^ | - Not serious | - Serious^5^ | - None | - 0.443 smaller in the PT group [from -0.933 to 0.047; p=0.076] | - ⨁◯◯◯ | - **VERY LOW** |
| - **U4** | 1 | - Serious^1^ | - Serious^2^ | - Not serious | - Serious^5^ | - None | - -0.033 smaller in the PT group [from -0.325 to 0.259; p=0.823] | - ⨁◯◯◯ | - **VERY LOW** |
| - **U5** | 1 | - Serious^1^ | - Not serious | - Not serious | - Serious^5^ | - None | - -0.100 smaller in the PT group [from -0.229 to 0.029; p=0.127] | - ⨁⨁◯◯ | - **LOW** |
| - **U6** | 3 | - Serious^1^ | - Serious^2^ | - Not serious | - Not serious | - None | - -0.174 smaller in the PT group [from -0.264 to -0.084; p=0.000] | - ⨁⨁⨁◯ | - **MODERATE^3^** |
| - **U7** | 1 | - Serious^1^ | - Not serious | - Not serious | - Serious^5^ | - None | - -0.305 smaller in the PT group [from -0.585 to -0.024; p=0.033] | - ⨁⨁◯◯ | - **LOW** |
| - **L1** | 3 | - Serious^1^ | - Serious^2^ | - Not serious | - Not serious | - None | - -0.117 smaller in the PT group [from -0.167 to -0.067; p=0.000] | - ⨁⨁⨁◯ | - **MODERATE^3^** |
| - **L2** | 3 | - Serious^1^ | - Serious^2^ | - Not serious | - Not serious | - None | - -0.341 smaller in the PT group [from -0.447 to -0.236; p=0.000] | - ⨁⨁⨁◯ | - **MODERATE^3^** |
| - **L3** | 1 | - Serious^1^ | - Serious^2^ | - Not serious | - Serious^5^ | - None | - -0.395 smaller in the PT group [from -0.786 to -0.003; p=0.048] | - ⨁◯◯◯ | - **VERY LOW** |
| - **L4** | 1 | - Serious^1^ | - Serious^2^ | - Not serious | - Serious^5^ | - None | - -0.034 smaller in the PT group [from -0.326 to 0.258; p=0.820] | - ⨁◯◯◯ | - **VERY LOW** |
| - **L5** | 1 | - Serious^1^ | - Not serious | - Not serious | - Serious^5^ | - None | - -0.100 smaller in the PT group [from -0.238 to 0.038; p=0.155] | - ⨁⨁◯◯ | - **LOW** |
| - **L6** | 3 | - Serious^1^ | - Serious^2^ | - Not serious | - Not serious | - None | - -0.136 smaller in the PT group [from -0.223 to -0.050; p=0.002] | - ⨁⨁⨁◯ | - **MODERATE^3^** |
| - **L7** | 1 | - Serious^1^ | - Serious^2^ | - Not serious | - Serious^5^ | - None | - -0.400 smaller in the PT group [from -0.679 to -0.121; p=0.005] | - ⨁◯◯◯ | - **VERY LOW** |
|  | - **Differences in permanent teeth dimensions between preterm and full-term children - Buccolingual dimensions** | | | | | | | | |
| - **U1** | 1 | - Serious^1^ | - Not serious | - Not serious | - Serious^5^ | - None | - -0.234 smaller in the PT group [from -0.523 to 0.055; p=0.112] | - ⨁⨁◯◯ | - **LOW** |
| - **U2** | 1 | - Serious^1^ | - Not serious | - Not serious | - Serious^5^ | - None | - 0.100 smaller in the PT group [from -0.249 to 0.449; p=0.574] | - ⨁⨁◯◯ | - **LOW** |
| - **U6** | 2 | - Serious^1^ | - Serious^2^ | - Not serious | - Not serious | - None | - -0.049 smaller in the PT group [from -0.112 to 0.014; p=0.130] | - ⨁⨁⨁◯ | - **MODERATE^3^** |
| - **L1** | 1 | - Serious^1^ | - Not serious | - Not serious | - Serious^5^ | - None | - -0.248 smaller in the PT group [from -0.479 to -0.017; p=0.035] | - ⨁⨁◯◯ | - **LOW** |
| - **L2** | 1 | - Serious^1^ | - Not serious | - Not serious | - Serious^5^ | - None | - -0.131 smaller in the PT group [from -0.447 to 0.185; p=0.418] | - ⨁⨁◯◯ | - **LOW** |
| - **L6** | 2 | - Serious^1^ | - Serious^2^ | - Not serious | - Not serious | - None | - -0.130 smaller in the PT group [from -0.218 to -0.042; p=0.004] | - ⨁⨁⨁◯ | - **MODERATE^3^** |

- CI: Confidence Interval; PT: preterm; WMD: Weighted Mean Difference

^1^Information comes from studies with serious risk of bias that adjusted for major confounders, but as they are not randomized a risk of unmeasured confounding might still exist; ^2^I-squared may represent substantial heterogeneity or the prediction interval includes the zero value indicating the presence of heterogeneity; ^3^Assessement was rated up once because of the presence of a dose response effect of the gestational age on the differences in permanent teeth dimensions between preterm and full-term children; ^4^The information comes from a limited number of individuals.
